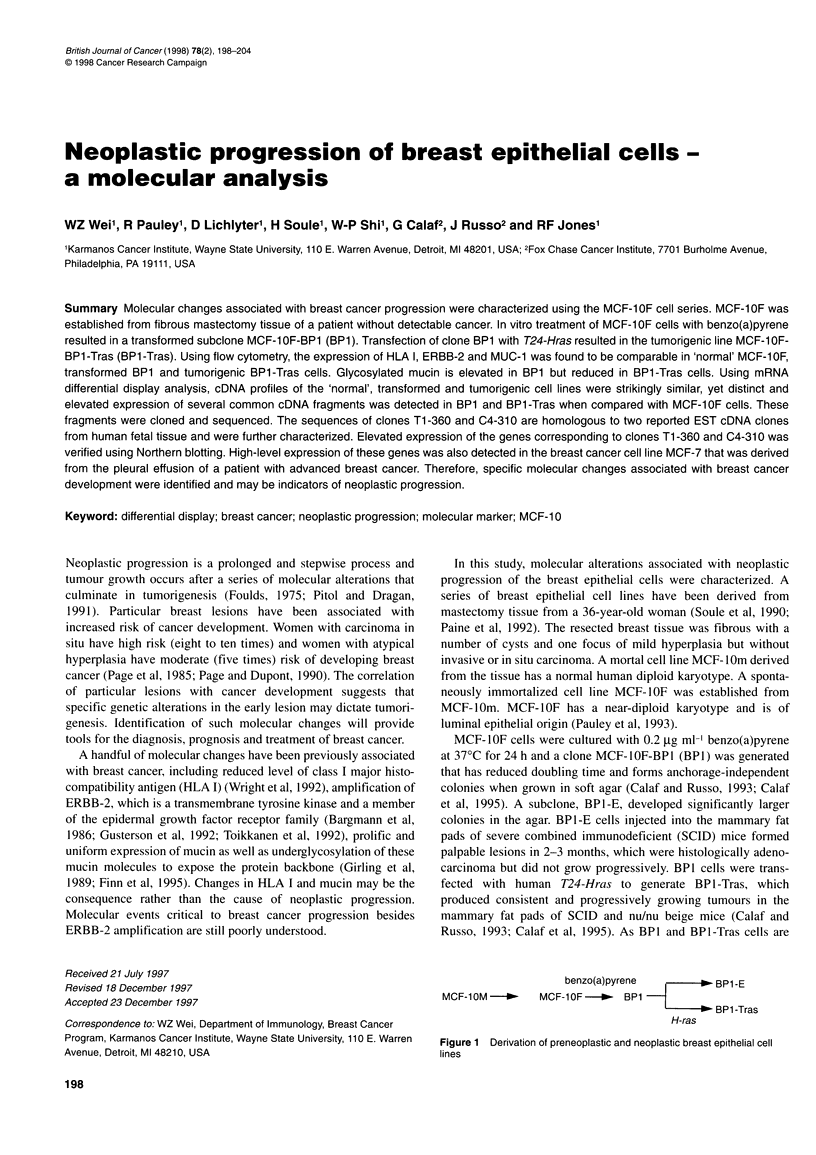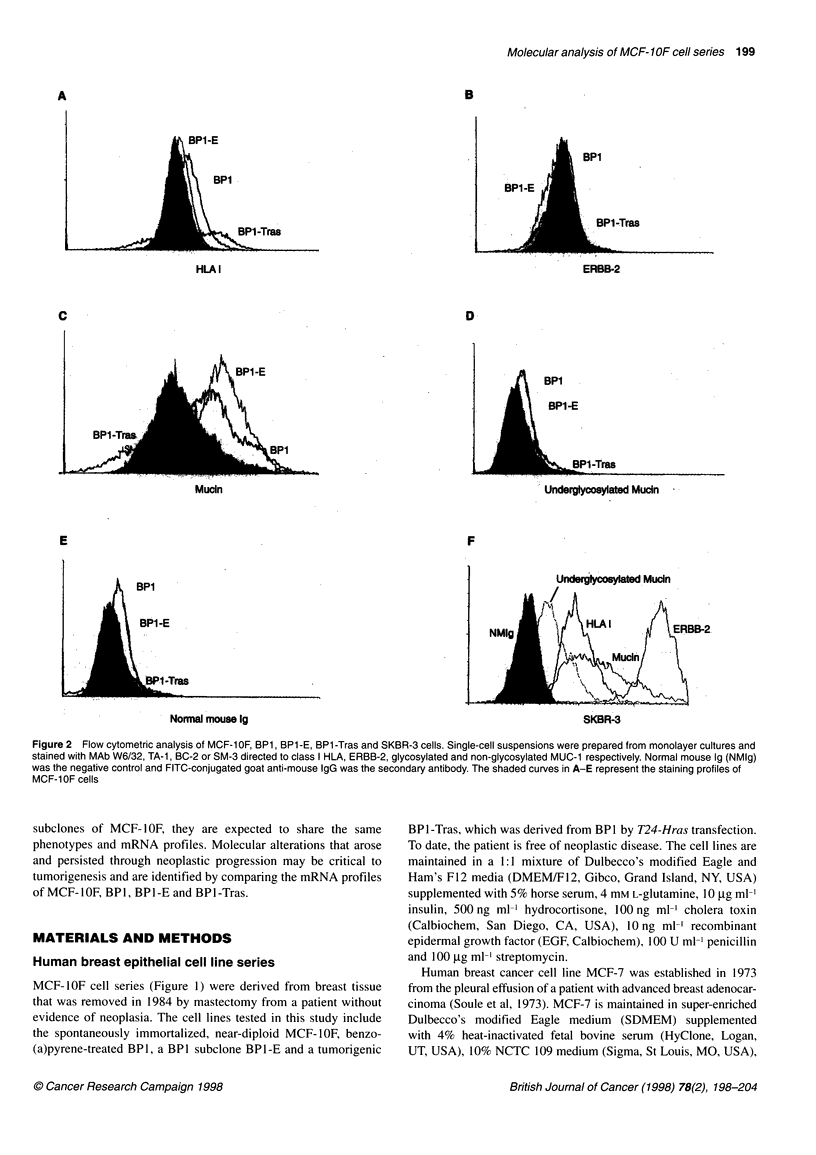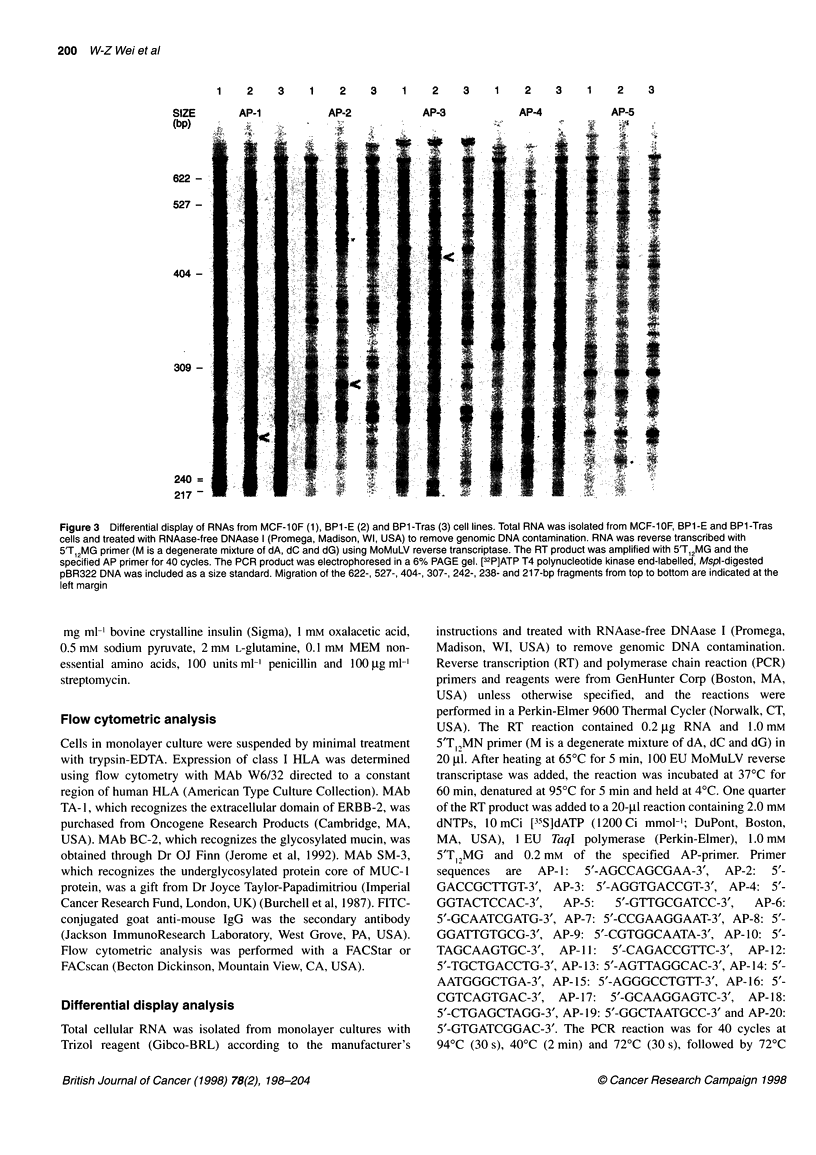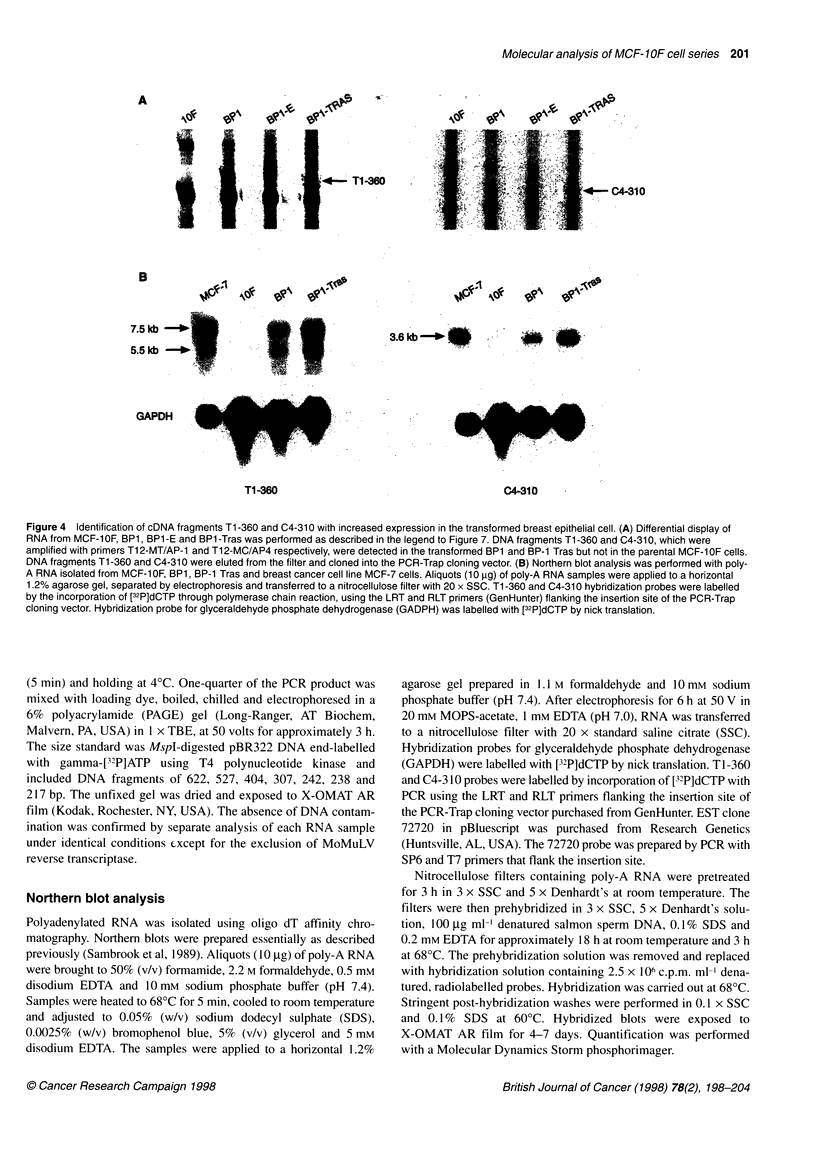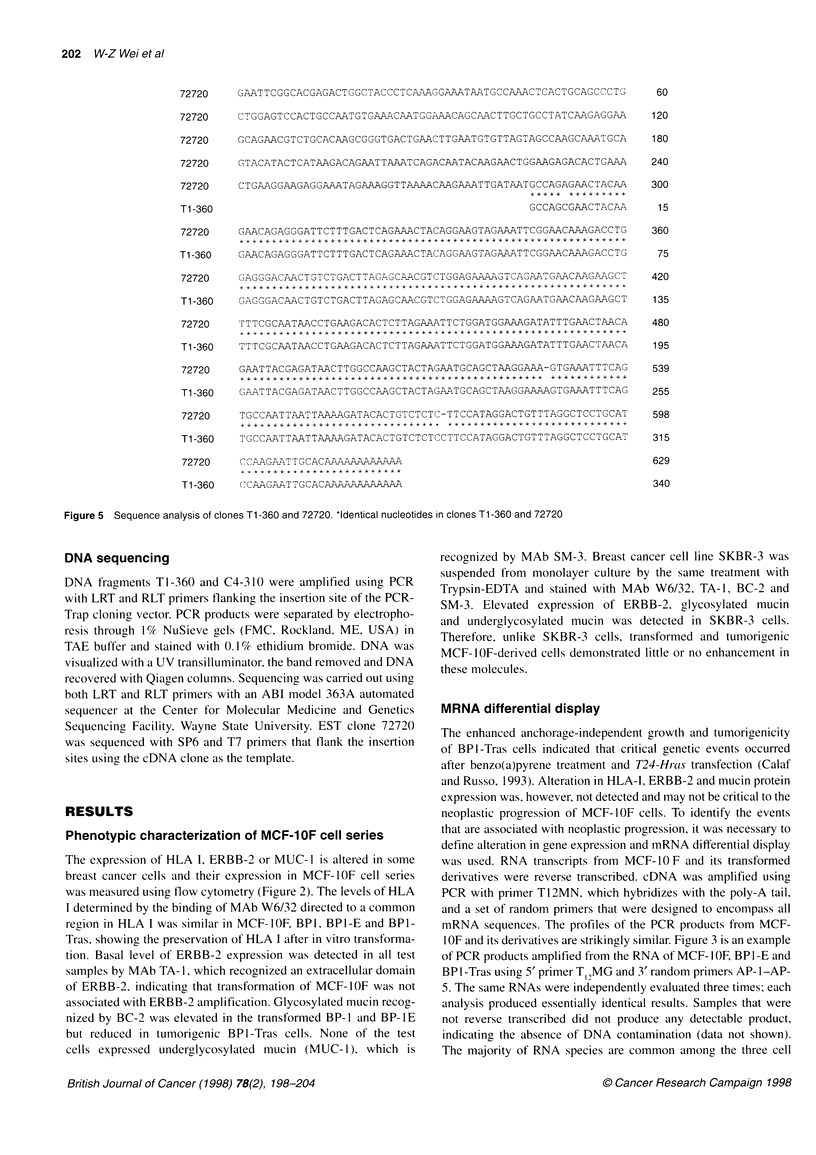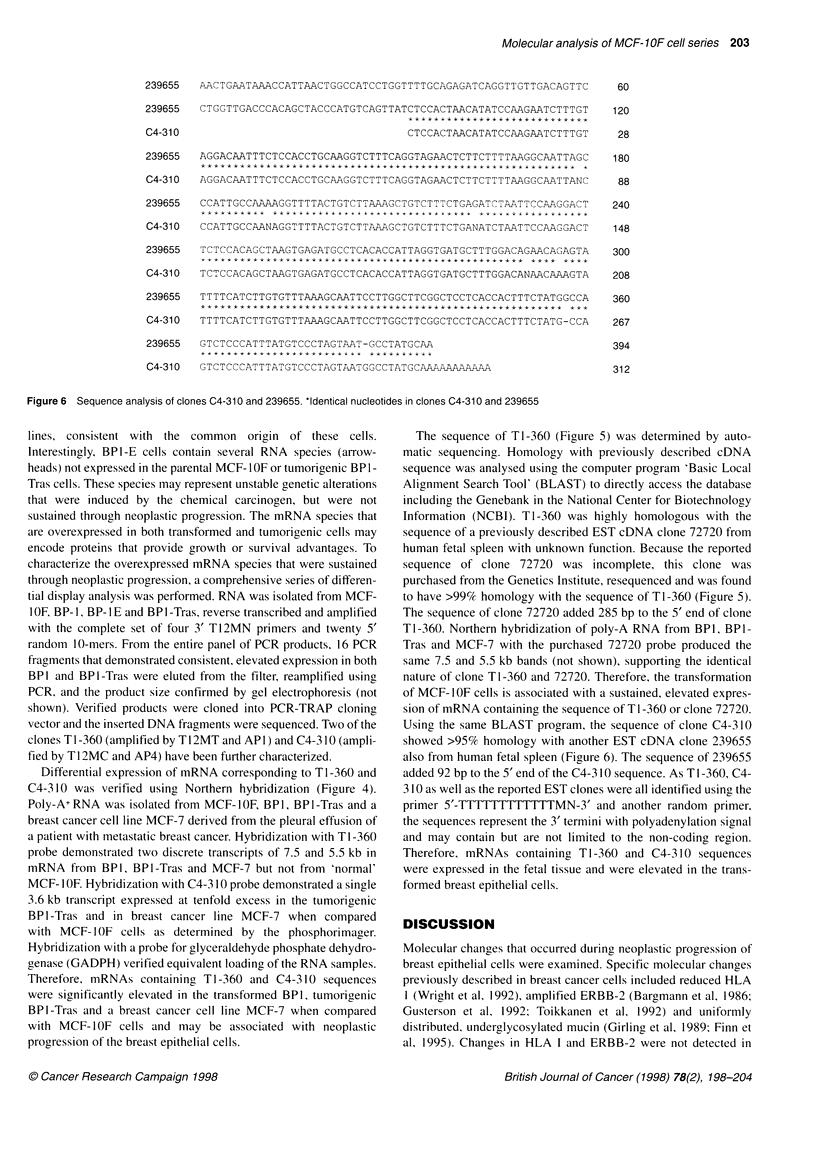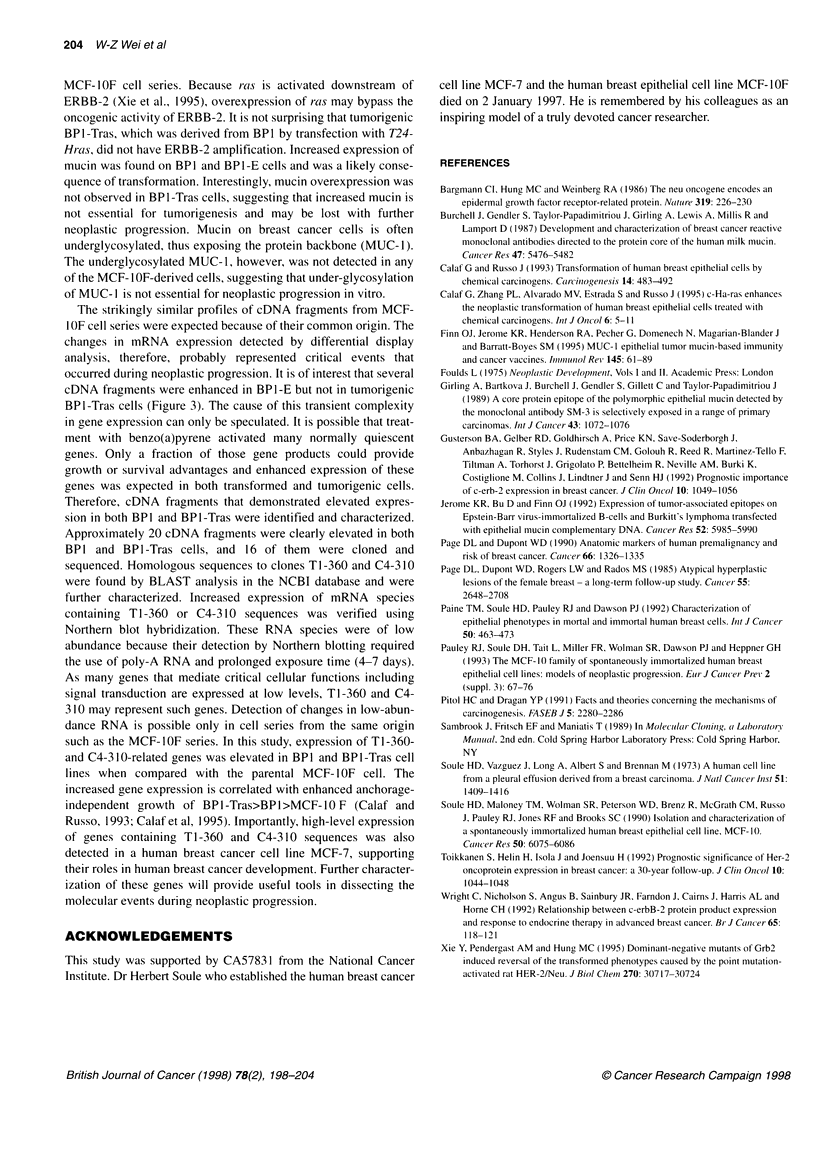# Neoplastic progression of breast epithelial cells--a molecular analysis.

**DOI:** 10.1038/bjc.1998.464

**Published:** 1998-07

**Authors:** W. Z. Wei, R. Pauley, D. Lichlyter, H. Soule, W. P. Shi, G. Calaf, J. Russo, R. F. Jones

**Affiliations:** Karmanos Cancer Institute, Wayne State University, Detroit, MI 48201, USA.

## Abstract

**Images:**


*British Journal of Cancer* (1998) **78**(2), 198–204
© 1998 Cancer Research Campaign

### Neoplastic progression of breast epithelial cells – a molecular analysis

**WZ Wei[1], R Pauley[1], D Lichlyter[1], H Soule[1], W-P Shi[1], G Calaf[2], J Russo[2] and RF Jones[1]**

[1]Karmanos Cancer Institute, Wayne State University, 110 E. Warren Avenue, Detroit, MI 48201, USA; [2]Fox Chase Cancer Institute, 7701 Burholme Avenue, Philadelphia, PA 19111, USA

**Summary** Molecular changes associated with breast cancer progression were characterized using the MCF-10F cell series. MCF-10F was established from fibrous mastectomy tissue of a patient without detectable cancer. In vitro treatment of MCF-10F cells with benzo(a)pyrene resulted in a transformed subclone MCF-10F-BP1 (BP1). Transfection of clone BP1 with *T24-Hras* resulted in the tumorigenic line MCF-10F-BP1-Tras (BP1-Tras). Using flow cytometry, the expression of HLA I, ERBB-2 and MUC-1 was found to be comparable in 'normal' MCF-10F, transformed BP1 and tumorigenic BP1-Tras cells. Glycosylated mucin is elevated in BP1 but reduced in BP1-Tras cells. Using mRNA differential display analysis, cDNA profiles of the 'normal', transformed and tumorigenic cell lines were strikingly similar, yet distinct and elevated expression of several common cDNA fragments was detected in BP1 and BP1-Tras when compared with MCF-10F cells. These fragments were cloned and sequenced. The sequences of clones T1-360 and C4-310 are homologous to two reported EST cDNA clones from human fetal tissue and were further characterized. Elevated expression of the genes corresponding to clones T1-360 and C4-310 was verified using Northern blotting. High-level expression of these genes was also detected in the breast cancer cell line MCF-7 that was derived from the pleural effusion of a patient with advanced breast cancer. Therefore, specific molecular changes associated with breast cancer development were identified and may be indicators of neoplastic progression.

**Keyword:** differential display; breast cancer; neoplastic progression; molecular marker; MCF-10

Neoplastic progression is a prolonged and stepwise process and tumour growth occurs after a series of molecular alterations that culminate in tumorigenesis (Foulds, 1975; Pitol and Dragan, 1991). Particular breast lesions have been associated with increased risk of cancer development. Women with carcinoma in situ have high risk (eight to ten times) and women with atypical hyperplasia have moderate (five times) risk of developing breast cancer (Page et al, 1985; Page and Dupont, 1990). The correlation of particular lesions with cancer development suggests that specific genetic alterations in the early lesion may dictate tumorigenesis. Identification of such molecular changes will provide tools for the diagnosis, prognosis and treatment of breast cancer.

A handful of molecular changes have been previously associated with breast cancer, including reduced level of class I major histocompatibility antigen (HLA I) (Wright et al, 1992), amplification of ERBB-2, which is a transmembrane tyrosine kinase and a member of the epidermal growth factor receptor family (Bargmann et al, 1986; Gusterson et al, 1992; Toikkanen et al, 1992), prolific and uniform expression of mucin as well as underglycosylation of these mucin molecules to expose the protein backbone (Girling et al, 1989; Finn et al, 1995). Changes in HLA I and mucin may be the consequence rather than the cause of neoplastic progression. Molecular events critical to breast cancer progression besides ERBB-2 amplification are still poorly understood.

In this study, molecular alterations associated with neoplastic progression of the breast epithelial cells were characterized. A series of breast epithelial cell lines have been derived from mastectomy tissue from a 36-year-old woman (Soule et al, 1990; Paine et al, 1992). The resected breast tissue was fibrous with a number of cysts and one focus of mild hyperplasia but without invasive or in situ carcinoma. A mortal cell line MCF-10m derived from the tissue has a normal human diploid karyotype. A spontaneously immortalized cell line MCF-10F was established from MCF-10m. MCF-10F has a near-diploid karyotype and is of luminal epithelial origin (Pauley et al, 1993).

MCF-10F cells were cultured with 0.2 μg $ml^{-1}$ benzo(a)pyrene at 37°C for 24 h and a clone MCF-10F-BP1 (BP1) was generated that has reduced doubling time and forms anchorage-independent colonies when grown in soft agar (Calaf and Russo, 1993; Calaf et al, 1995). A subclone, BP1-E, developed significantly larger colonies in the agar. BP1-E cells injected into the mammary fat pads of severe combined immunodeficient (SCID) mice formed palpable lesions in 2–3 months, which were histologically adenocarcinoma but did not grow progressively. BP1 cells were transfected with human *T24-Hras* to generate BP1-Tras, which produced consistent and progressively growing tumours in the mammary fat pads of SCID and nu/nu beige mice (Calaf and Russo, 1993; Calaf et al, 1995). As BP1 and BP1-Tras cells are

**Figure 1** Derivation of preneoplastic and neoplastic breast epithelial cell lines

*Received 21 July 1997*
*Revised 18 December 1997*
*Accepted 23 December 1997*

*Correspondence to:* WZ Wei, Department of Immunology, Breast Cancer Program, Karmanos Cancer Institute, Wayne State University, 110 E. Warren Avenue, Detroit, MI 48210, USA

**Figure 2** Flow cytometric analysis of MCF-10F, BP1, BP1-E, BP1-Tras and SKBR-3 cells. Single-cell suspensions were prepared from monolayer cultures and stained with MAb W6/32, TA-1, BC-2 or SM-3 directed to class I HLA, ERBB-2, glycosylated and non-glycosylated MUC-1 respectively. Normal mouse Ig (NMIg) was the negative control and FITC-conjugated goat anti-mouse IgG was the secondary antibody. The shaded curves in **A**–**E** represent the staining profiles of MCF-10F cells subclones of MCF-10F, they are expected to share the same phenotypes and mRNA profiles. Molecular alterations that arose and persisted through neoplastic progression may be critical to tumorigenesis and are identified by comparing the mRNA profiles of MCF-10F, BP1, BP1-E and BP1-Tras.

### MATERIALS AND METHODS

### Human breast epithelial cell line series

MCF-10F cell series (Figure 1) were derived from breast tissue that was removed in 1984 by mastectomy from a patient without evidence of neoplasia. The cell lines tested in this study include the spontaneously immortalized, near-diploid MCF-10F, benzo-(a)pyrene-treated BP1, a BP1 subclone BP1-E and a tumorigenic BP1-Tras, which was derived from BP1 by *T24-Hras* transfection. To date, the patient is free of neoplastic disease. The cell lines are maintained in a 1:1 mixture of Dulbecco's modified Eagle and Ham's F12 media (DMEM/F12, Gibco, Grand Island, NY, USA) supplemented with 5% horse serum, 4 mM L-glutamine, 10 $\mu$g ml$^{-1}$ insulin, 500 ng ml$^{-1}$ hydrocortisone, 100 ng ml$^{-1}$ cholera toxin (Calbiochem, San Diego, CA, USA), 10 ng ml$^{-1}$ recombinant epidermal growth factor (EGF, Calbiochem), 100 U ml$^{-1}$ penicillin and 100 $\mu$g ml$^{-1}$ streptomycin.

Human breast cancer cell line MCF-7 was established in 1973 from the pleural effusion of a patient with advanced breast adenocarcinoma (Soule et al, 1973). MCF-7 is maintained in super-enriched Dulbecco's modified Eagle medium (SDMEM) supplemented with 4% heat-inactivated fetal bovine serum (HyClone, Logan, UT, USA), 10% NCTC 109 medium (Sigma, St Louis, MO, USA),

© Cancer Research Campaign 1998

**Figure 3** Differential display of RNAs from MCF-10F (1), BP1-E (2) and BP1-Tras (3) cell lines. Total RNA was isolated from MCF-10F, BP1-E and BP1-Tras cells and treated with RNAase-free DNAase I (Promega, Madison, WI, USA) to remove genomic DNA contamination. RNA was reverse transcribed with $5'T_{12}MG$ primer (M is a degenerate mixture of dA, dC and dG) using MoMuLV reverse transcriptase. The RT product was amplified with $5'T_{12}MG$ and the specified AP primer for 40 cycles. The PCR product was electrophoresed in a 6% PAGE gel. [$^{32}$P]ATP T4 polynucleotide kinase end-labelled, *Msp*I-digested pBR322 DNA was included as a size standard. Migration of the 622-, 527-, 404-, 307-, 242-, 238- and 217-bp fragments from top to bottom are indicated at the left margin mg ml$^{-1}$ bovine crystalline insulin (Sigma), 1 mM oxalacetic acid, 0.5 mM sodium pyruvate, 2 mM L-glutamine, 0.1 mM MEM non-essential amino acids, 100 units ml$^{-1}$ penicillin and 100 μg ml$^{-1}$ streptomycin.

### Flow cytometric analysis

Cells in monolayer culture were suspended by minimal treatment with trypsin-EDTA. Expression of class I HLA was determined using flow cytometry with MAb W6/32 directed to a constant region of human HLA (American Type Culture Collection). MAb TA-1, which recognizes the extracellular domain of ERBB-2, was purchased from Oncogene Research Products (Cambridge, MA, USA). MAb BC-2, which recognizes the glycosylated mucin, was obtained through Dr OJ Finn (Jerome et al, 1992). MAb SM-3, which recognizes the underglycosylated protein core of MUC-1 protein, was a gift from Dr Joyce Taylor-Papadimitriou (Imperial Cancer Research Fund, London, UK) (Burchell et al, 1987). FITC-conjugated goat anti-mouse IgG was the secondary antibody (Jackson ImmunoResearch Laboratory, West Grove, PA, USA). Flow cytometric analysis was performed with a FACStar or FACscan (Becton Dickinson, Mountain View, CA, USA).

### Differential display analysis

Total cellular RNA was isolated from monolayer cultures with Trizol reagent (Gibco-BRL) according to the manufacturer's instructions and treated with RNAase-free DNAase I (Promega, Madison, WI, USA) to remove genomic DNA contamination. Reverse transcription (RT) and polymerase chain reaction (PCR) primers and reagents were from GenHunter Corp (Boston, MA, USA) unless otherwise specified, and the reactions were performed in a Perkin-Elmer 9600 Thermal Cycler (Norwalk, CT, USA). The RT reaction contained 0.2 μg RNA and 1.0 mM $5'T_{12}MN$ primer (M is a degenerate mixture of dA, dC and dG) in 20 μl. After heating at 65°C for 5 min, 100 EU MoMuLV reverse transcriptase was added, the reaction was incubated at 37°C for 60 min, denatured at 95°C for 5 min and held at 4°C. One quarter of the RT product was added to a 20-μl reaction containing 2.0 mM dNTPs, 10 mCi [$^{35}$S]dATP (1200 Ci mmol$^{-1}$; DuPont, Boston, MA, USA), 1 EU *Taq*I polymerase (Perkin-Elmer), 1.0 mM $5'T_{12}MG$ and 0.2 mM of the specified AP-primer. Primer sequences are AP-1: 5′-AGCCAGCGAA-3′, AP-2: 5′-GACCGCTTGT-3′, AP-3: 5′-AGGTGACCGT-3′, AP-4: 5′-GGTACTCCAC-3′, AP-5: 5′-GTTGCGATCC-3′, AP-6: 5′-GCAATCGATG-3′, AP-7: 5′-CCGAAGGAAT-3′, AP-8: 5′-GGATTGTGCG-3′, AP-9: 5′-CGTGGCAATA-3′, AP-10: 5′-TAGCAAGTGC-3′, AP-11: 5′-CAGACCGTTC-3′, AP-12: 5′-TGCTGACCTG-3′, AP-13: 5′-AGTTAGGCAC-3′, AP-14: 5′-AATGGGCTGA-3′, AP-15: 5′-AGGGCCTGTT-3′, AP-16: 5′-CGTCAGTGAC-3′, AP-17: 5′-GCAAGGAGTC-3′, AP-18: 5′-CTGAGCTAGG-3′, AP-19: 5′-GGCTAATGCC-3′ and AP-20: 5′-GTGATCGGAC-3′. The PCR reaction was for 40 cycles at 94°C (30 s), 40°C (2 min) and 72°C (30 s), followed by 72°C

© Cancer Research Campaign 1998

**Figure 4** Identification of cDNA fragments T1-360 and C4-310 with increased expression in the transformed breast epithelial cell. (**A**) Differential display of RNA from MCF-10F, BP1, BP1-E and BP1-Tras was performed as described in the legend to Figure 7. DNA fragments T1-360 and C4-310, which were amplified with primers T12-MT/AP-1 and T12-MC/AP4 respectively, were detected in the transformed BP1 and BP-1 Tras but not in the parental MCF-10F cells. DNA fragments T1-360 and C4-310 were eluted from the filter and cloned into the PCR-Trap cloning vector. (**B**) Northern blot analysis was performed with poly-A RNA isolated from MCF-10F, BP1, BP-1 Tras and breast cancer cell line MCF-7 cells. Aliquots (10 µg) of poly-A RNA samples were applied to a horizontal 1.2% agarose gel, separated by electrophoresis and transferred to a nitrocellulose filter with 20 × SSC. T1-360 and C4-310 hybridization probes were labelled by the incorporation of [$^{32}$P]dCTP through polymerase chain reaction, using the LRT and RLT primers (GenHunter) flanking the insertion site of the PCR-Trap cloning vector. Hybridization probe for glyceraldehyde phosphate dehydrogenase (GADPH) was labelled with [$^{32}$P]dCTP by nick translation.

(5 min) and holding at 4°C. One-quarter of the PCR product was mixed with loading dye, boiled, chilled and electrophoresed in a 6% polyacrylamide (PAGE) gel (Long-Ranger, AT Biochem, Malvern, PA, USA) in 1 × TBE, at 50 volts for approximately 3 h. The size standard was *Msp*I-digested pBR322 DNA end-labelled with gamma-[$^{32}$P]ATP using T4 polynucleotide kinase and included DNA fragments of 622, 527, 404, 307, 242, 238 and 217 bp. The unfixed gel was dried and exposed to X-OMAT AR film (Kodak, Rochester, NY, USA). The absence of DNA contamination was confirmed by separate analysis of each RNA sample under identical conditions except for the exclusion of MoMuLV reverse transcriptase.

### Northern blot analysis

Polyadenylated RNA was isolated using oligo dT affinity chromatography. Northern blots were prepared essentially as described previously (Sambrook et al, 1989). Aliquots (10 µg) of poly-A RNA were brought to 50% (v/v) formamide, 2.2 M formaldehyde, 0.5 mM disodium EDTA and 10 mM sodium phosphate buffer (pH 7.4). Samples were heated to 68°C for 5 min, cooled to room temperature and adjusted to 0.05% (w/v) sodium dodecyl sulphate (SDS), 0.0025% (w/v) bromophenol blue, 5% (v/v) glycerol and 5 mM disodium EDTA. The samples were applied to a horizontal 1.2% agarose gel prepared in 1.1 M formaldehyde and 10 mM sodium phosphate buffer (pH 7.4). After electrophoresis for 6 h at 50 V in 20 mM MOPS-acetate, 1 mM EDTA (pH 7.0), RNA was transferred to a nitrocellulose filter with 20 × standard saline citrate (SSC). Hybridization probes for glyceraldehyde phosphate dehydrogenase (GAPDH) were labelled with [$^{32}$P]dCTP by nick translation. T1-360 and C4-310 probes were labelled by incorporation of [$^{32}$P]dCTP with PCR using the LRT and RLT primers flanking the insertion site of the PCR-Trap cloning vector purchased from GenHunter. EST clone 72720 in pBluescript was purchased from Research Genetics (Huntsville, AL, USA). The 72720 probe was prepared by PCR with SP6 and T7 primers that flank the insertion site.

Nitrocellulose filters containing poly-A RNA were pretreated for 3 h in 3 × SSC and 5 × Denhardt's at room temperature. The filters were then prehybridized in 3 × SSC, 5 × Denhardt's solution, 100 µg ml$^{-1}$ denatured salmon sperm DNA, 0.1% SDS and 0.2 mM EDTA for approximately 18 h at room temperature and 3 h at 68°C. The prehybridization solution was removed and replaced with hybridization solution containing 2.5 × 10$^{6}$ c.p.m. ml$^{-1}$ denatured, radiolabelled probes. Hybridization was carried out at 68°C. Stringent post-hybridization washes were performed in 0.1 × SSC and 0.1% SDS at 60°C. Hybridized blots were exposed to X-OMAT AR film for 4–7 days. Quantification was performed with a Molecular Dynamics Storm phosphorimager.

© Cancer Research Campaign 1998

```
72720    GAATTCGGCACGAGACTGGCTACCCTCAAAGGAAATAATGCCAAACTCACTGCAGCCCTG   60

72720    CTGGAGTCCACTGCCAATGTGAAACAATGGAAACAGCAACTTGCTGCCTATCAAGAGGAA   120

72720    GCAGAACGTCTGCACAAGCGGGTGACTGAACTTGAATGTGTTAGTAGCCAAGCAAATGCA   180

72720    GTACATACTCATAAGACAGAATTAAATCAGACAATACAAGAACTGGAAGAGACACTGAAA   240

72720    CTGAAGGAAGAGGAAATAGAAAGGTTAAAACAAGAAATTGATAATGCCAGAGAACTACAA   300
                                                      ***** *********
T1-360                                                GCCAGCGAACTACAA   15

72720    GAACAGAGGGATTCTTTGACTCAGAAACTACAGGAAGTAGAAATTCGGAACAAAGACCTG   360
         ************************************************************
T1-360   GAACAGAGGGATTCTTTGACTCAGAAACTACAGGAAGTAGAAATTCGGAACAAAGACCTG   75

72720    GAGGGACAACTGTCTGACTTAGAGCAACGTCTGGAGAAAAGTCAGAATGAACAAGAAGCT   420

         ************************************************************

T1-360   GAGGGACAACTGTCTGACTTAGAGCAACGTCTGGAGAAAAGTCAGAATGAACAAGAAGCT   135

72720    TTTCGCAATAACCTGAAGACACTCTTAGAAATTCTGGATGGAAAGATATTTGAACTAACA   480
         ************************************************************
T1-360   TTTCGCAATAACCTGAAGACACTCTTAGAAATTCTGGATGGAAAGATATTTGAACTAACA   195

72720    GAATTACGAGATAACTTGGCCAAGCTACTAGAATGCAGCTAAGGAAA-GTGAAATTTCAG   539
         *********************************************** ************
T1-360   GAATTACGAGATAACTTGGCCAAGCTACTAGAATGCAGCTAAGGAAAAGTGAAATTTCAG   255

72720    TGCCAATTAATTAAAAGATACACTGTCTCTC-TTCCATAGGACTGTTTAGGCTCCTGCAT   598
         ******************************* ****************************
T1-360   TGCCAATTAATTAAAAGATACACTGTCTCTCCTTCCATAGGACTGTTTAGGCTCCTGCAT   315

72720    CCAAGAATTGCACAAAAAAAAAAAA                                      629

         *************************

T1-360   CCAAGAATTGCACAAAAAAAAAAAA                                      340

```

**Figure 5** Sequence analysis of clones T1-360 and 72720. *Identical nucleotides in clones T1-360 and 72720

### DNA sequencing

DNA fragments T1-360 and C4-310 were amplified using PCR with LRT and RLT primers flanking the insertion site of the PCR-Trap cloning vector. PCR products were separated by electrophoresis through 1% NuSieve gels (FMC, Rockland, ME, USA) in TAE buffer and stained with 0.1% ethidium bromide. DNA was visualized with a UV transilluminator, the band removed and DNA recovered with Qiagen columns. Sequencing was carried out using both LRT and RLT primers with an ABI model 363A automated sequencer at the Center for Molecular Medicine and Genetics Sequencing Facility, Wayne State University. EST clone 72720 was sequenced with SP6 and T7 primers that flank the insertion sites using the cDNA clone as the template.

### RESULTS

### Phenotypic characterization of MCF-10F cell series

The expression of HLA I, ERBB-2 or MUC-1 is altered in some breast cancer cells and their expression in MCF-10F cell series was measured using flow cytometry (Figure 2). The levels of HLA I determined by the binding of MAb W6/32 directed to a common region in HLA I was similar in MCF-10F, BP1, BP1-E and BP1-Tras, showing the preservation of HLA I after in vitro transformation. Basal level of ERBB-2 expression was detected in all test samples by MAb TA-1, which recognized an extracellular domain of ERBB-2, indicating that transformation of MCF-10F was not associated with ERBB-2 amplification. Glycosylated mucin recognized by BC-2 was elevated in the transformed BP-1 and BP-1E but reduced in tumorigenic BP1-Tras cells. None of the test cells expressed underglycosylated mucin (MUC-1), which is recognized by MAb SM-3. Breast cancer cell line SKBR-3 was suspended from monolayer culture by the same treatment with Trypsin-EDTA and stained with MAb W6/32, TA-1, BC-2 and SM-3. Elevated expression of ERBB-2, glycosylated mucin and underglycosylated mucin was detected in SKBR-3 cells. Therefore, unlike SKBR-3 cells, transformed and tumorigenic MCF-10F-derived cells demonstrated little or no enhancement in these molecules.

### MRNA differential display

The enhanced anchorage-independent growth and tumorigenicity of BP1-Tras cells indicated that critical genetic events occurred after benzo(a)pyrene treatment and *T24-Hras* transfection (Calaf and Russo, 1993). Alteration in HLA-I, ERBB-2 and mucin protein expression was, however, not detected and may not be critical to the neoplastic progression of MCF-10F cells. To identify the events that are associated with neoplastic progression, it was necessary to define alteration in gene expression and mRNA differential display was used. RNA transcripts from MCF-10 F and its transformed derivatives were reverse transcribed. cDNA was amplified using PCR with primer T12MN, which hybridizes with the poly-A tail, and a set of random primers that were designed to encompass all mRNA sequences. The profiles of the PCR products from MCF-10F and its derivatives are strikingly similar. Figure 3 is an example of PCR products amplified from the RNA of MCF-10F, BP1-E and BP1-Tras using 5′ primer $T_{12}$MG and 3′ random primers AP-1–AP-5. The same RNAs were independently evaluated three times; each analysis produced essentially identical results. Samples that were not reverse transcribed did not produce any detectable product, indicating the absence of DNA contamination (data not shown). The majority of RNA species are common among the three cell

© Cancer Research Campaign 1998

```
239655   AACTGAATAAACCATTAACTGGCCATCCTGGTTTTGCAGAGATCAGGTTGTTGACAGTTC   60

239655   CTGGTTGACCCACAGCTACCCATGTCAGTTATCTCCACTAACATATCCAAGAATCTTTGT   120
                                         ****************************
C4-310                                   CTCCACTAACATATCCAAGAATCTTTGT   28

239655   AGGACAATTTCTCCACCTGCAAGGTCTTTCAGGTAGAACTCTTCTTTTAAGGCAATTAGC   180
         ********************************************************** *
C4-310   AGGACAATTTCTCCACCTGCAAGGTCTTTCAGGTAGAACTCTTCTTTTAAGGCAATTANC   88

239655   CCATTGCCAAAAGGTTTTACTGTCTTAAAGCTGTCTTTCTGAGATCTAATTCCAAGGACT   240
         ********** ******************************* *****************
C4-310   CCATTGCCAANAGGTTTTACTGTCTTAAAGCTGTCTTTCTGANATCTAATTCCAAGGACT   148

239655   TCTCCACAGCTAAGTGAGATGCCTCACACCATTAGGTGATGCTTTGGACAGAACAGAGTA   300
         ************************************************** **** ****
C4-310   TCTCCACAGCTAAGTGAGATGCCTCACACCATTAGGTGATGCTTTGGACANAACAAAGTA   208

239655   TTTTCATCTTGTGTTTAAAGCAATTCCTTGGCTTCGGCTCCTCACCACTTTCTATGGCCA   360
         ******************************************************** ***
C4-310   TTTTCATCTTGTGTTTAAAGCAATTCCTTGGCTTCGGCTCCTCACCACTTTCTATG-CCA   267

239655   GTCTCCCATTTATGTCCCTAGTAAT-GCCTATGCAA                           394

         ************************* **********

C4-310   GTCTCCCATTTATGTCCCTAGTAATGGCCTATGCAAAAAAAAAAAA                 312

```

**Figure 6** Sequence analysis of clones C4-310 and 239655. *Identical nucleotides in clones C4-310 and 239655

lines, consistent with the common origin of these cells. Interestingly, BP1-E cells contain several RNA species (arrowheads) not expressed in the parental MCF-10F or tumorigenic BP1-Tras cells. These species may represent unstable genetic alterations that were induced by the chemical carcinogen, but were not sustained through neoplastic progression. The mRNA species that are overexpressed in both transformed and tumorigenic cells may encode proteins that provide growth or survival advantages. To characterize the overexpressed mRNA species that were sustained through neoplastic progression, a comprehensive series of differential display analysis was performed. RNA was isolated from MCF-10F, BP-1, BP-1E and BP1-Tras, reverse transcribed and amplified with the complete set of four 3′ T12MN primers and twenty 5′ random 10-mers. From the entire panel of PCR products, 16 PCR fragments that demonstrated consistent, elevated expression in both BP1 and BP1-Tras were eluted from the filter, reamplified using PCR, and the product size confirmed by gel electrophoresis (not shown). Verified products were cloned into PCR-TRAP cloning vector and the inserted DNA fragments were sequenced. Two of the clones T1-360 (amplified by T12MT and AP1) and C4-310 (amplified by T12MC and AP4) have been further characterized.

Differential expression of mRNA corresponding to T1-360 and C4-310 was verified using Northern hybridization (Figure 4). Poly-A$^+$ RNA was isolated from MCF-10F, BP1, BP1-Tras and a breast cancer cell line MCF-7 derived from the pleural effusion of a patient with metastatic breast cancer. Hybridization with T1-360 probe demonstrated two discrete transcripts of 7.5 and 5.5 kb in mRNA from BP1, BP1-Tras and MCF-7 but not from 'normal' MCF-10F. Hybridization with C4-310 probe demonstrated a single 3.6 kb transcript expressed at tenfold excess in the tumorigenic BP1-Tras and in breast cancer line MCF-7 when compared with MCF-10F cells as determined by the phosphorimager. Hybridization with a probe for glyceraldehyde phosphate dehydrogenase (GADPH) verified equivalent loading of the RNA samples. Therefore, mRNAs containing T1-360 and C4-310 sequences were significantly elevated in the transformed BP1, tumorigenic BP1-Tras and a breast cancer cell line MCF-7 when compared with MCF-10F cells and may be associated with neoplastic progression of the breast epithelial cells.

The sequence of T1-360 (Figure 5) was determined by automatic sequencing. Homology with previously described cDNA sequence was analysed using the computer program 'Basic Local Alignment Search Tool' (BLAST) to directly access the database including the Genebank in the National Center for Biotechnology Information (NCBI). T1-360 was highly homologous with the sequence of a previously described EST cDNA clone 72720 from human fetal spleen with unknown function. Because the reported sequence of clone 72720 was incomplete, this clone was purchased from the Genetics Institute, resequenced and was found to have >99% homology with the sequence of T1-360 (Figure 5). The sequence of clone 72720 added 285 bp to the 5′ end of clone T1-360. Northern hybridization of poly-A RNA from BP1, BP1-Tras and MCF-7 with the purchased 72720 probe produced the same 7.5 and 5.5 kb bands (not shown), supporting the identical nature of clone T1-360 and 72720. Therefore, the transformation of MCF-10F cells is associated with a sustained, elevated expression of mRNA containing the sequence of T1-360 or clone 72720. Using the same BLAST program, the sequence of clone C4-310 showed >95% homology with another EST cDNA clone 239655 also from human fetal spleen (Figure 6). The sequence of 239655 added 92 bp to the 5′ end of the C4-310 sequence. As T1-360, C4-310 as well as the reported EST clones were all identified using the primer 5′-TTTTTTTTTTTTMN-3′ and another random primer, the sequences represent the 3′ termini with polyadenylation signal and may contain but are not limited to the non-coding region. Therefore, mRNAs containing T1-360 and C4-310 sequences were expressed in the fetal tissue and were elevated in the transformed breast epithelial cells.

### DISCUSSION

Molecular changes that occurred during neoplastic progression of breast epithelial cells were examined. Specific molecular changes previously described in breast cancer cells included reduced HLA 1 (Wright et al, 1992), amplified ERBB-2 (Bargmann et al, 1986; Gusterson et al, 1992; Toikkanen et al, 1992) and uniformly distributed, underglycosylated mucin (Girling et al, 1989; Finn et al, 1995). Changes in HLA I and ERBB-2 were not detected in

© Cancer Research Campaign 1998

MCF-10F cell series. Because *ras* is activated downstream of ERBB-2 (Xie et al., 1995), overexpression of *ras* may bypass the oncogenic activity of ERBB-2. It is not surprising that tumorigenic BP1-Tras, which was derived from BP1 by transfection with *T24-Hras*, did not have ERBB-2 amplification. Increased expression of mucin was found on BP1 and BP1-E cells and was a likely consequence of transformation. Interestingly, mucin overexpression was not observed in BP1-Tras cells, suggesting that increased mucin is not essential for tumorigenesis and may be lost with further neoplastic progression. Mucin on breast cancer cells is often underglycosylated, thus exposing the protein backbone (MUC-1). The underglycosylated MUC-1, however, was not detected in any of the MCF-10F-derived cells, suggesting that under-glycosylation of MUC-1 is not essential for neoplastic progression in vitro.

The strikingly similar profiles of cDNA fragments from MCF-10F cell series were expected because of their common origin. The changes in mRNA expression detected by differential display analysis, therefore, probably represented critical events that occurred during neoplastic progression. It is of interest that several cDNA fragments were enhanced in BP1-E but not in tumorigenic BP1-Tras cells (Figure 3). The cause of this transient complexity in gene expression can only be speculated. It is possible that treatment with benzo(a)pyrene activated many normally quiescent genes. Only a fraction of those gene products could provide growth or survival advantages and enhanced expression of these genes was expected in both transformed and tumorigenic cells. Therefore, cDNA fragments that demonstrated elevated expression in both BP1 and BP1-Tras were identified and characterized. Approximately 20 cDNA fragments were clearly elevated in both BP1 and BP1-Tras cells, and 16 of them were cloned and sequenced. Homologous sequences to clones T1-360 and C4-310 were found by BLAST analysis in the NCBI database and were further characterized. Increased expression of mRNA species containing T1-360 or C4-310 sequences was verified using Northern blot hybridization. These RNA species were of low abundance because their detection by Northern blotting required the use of poly-A RNA and prolonged exposure time (4–7 days). As many genes that mediate critical cellular functions including signal transduction are expressed at low levels, T1-360 and C4-310 may represent such genes. Detection of changes in low-abundance RNA is possible only in cell series from the same origin such as the MCF-10F series. In this study, expression of T1-360- and C4-310-related genes was elevated in BP1 and BP1-Tras cell lines when compared with the parental MCF-10F cell. The increased gene expression is correlated with enhanced anchorage-independent growth of BP1-Tras>BP1>MCF-10 F (Calaf and Russo, 1993; Calaf et al, 1995). Importantly, high-level expression of genes containing T1-360 and C4-310 sequences was also detected in a human breast cancer cell line MCF-7, supporting their roles in human breast cancer development. Further characterization of these genes will provide useful tools in dissecting the molecular events during neoplastic progression.

### ACKNOWLEDGEMENTS

This study was supported by CA57831 from the National Cancer Institute. Dr Herbert Soule who established the human breast cancer cell line MCF-7 and the human breast epithelial cell line MCF-10F died on 2 January 1997. He is remembered by his colleagues as an inspiring model of a truly devoted cancer researcher.

### REFERENCES

Bargmann CI, Hung MC and Weinberg RA (1986) The neu oncogene encodes an epidermal growth factor receptor-related protein. *Nature* **319**: 226–230

Burchell J, Gendler S, Taylor-Papadimitriou J, Girling A, Lewis A, Millis R and Lamport D (1987) Development and characterization of breast cancer reactive monoclonal antibodies directed to the protein core of the human milk mucin. *Cancer Res* **47**: 5476–5482

Calaf G and Russo J (1993) Transformation of human breast epithelial cells by chemical carcinogens. *Carcinogenesis* **14**: 483–492

Calaf G, Zhang PL, Alvarado MV, Estrada S and Russo J (1995) c-Ha-ras enhances the neoplastic transformation of human breast epithelial cells treated with chemical carcinogens. *Int J Oncol* **6**: 5–11

Finn OJ, Jerome KR, Henderson RA, Pecher G, Domenech N, Magarian-Blander J and Barratt-Boyes SM (1995) MUC-1 epithelial tumor mucin-based immunity and cancer vaccines. *Immunol Rev* **145**: 61–89

Foulds L (1975) *Neoplastic Development*, Vols I and II. Academic Press: London

Girling A, Bartkova J, Burchell J, Gendler S, Gillett C and Taylor-Papadimitriou J (1989) A core protein epitope of the polymorphic epithelial mucin detected by the monoclonal antibody SM-3 is selectively exposed in a range of primary carcinomas. *Int J Cancer* **43**: 1072–1076

Gusterson BA, Gelber RD, Goldhirsch A, Price KN, Save-Soderborgh J, Anbazhagan R, Styles J, Rudenstam CM, Golouh R, Reed R, Martinez-Tello F, Tiltman A, Torhorst J, Grigolato P, Bettelheim R, Neville AM, Burki K, Costiglione M, Collins J, Lindtner J and Senn HJ (1992) Prognostic importance of c-erb-2 expression in breast cancer. *J Clin Oncol* **10**: 1049–1056

Jerome KR, Bu D and Finn OJ (1992) Expression of tumor-associated epitopes on Epstein-Barr virus-immortalized B-cells and Burkitt's lymphoma transfected with epithelial mucin complementary DNA. *Cancer Res* **52**: 5985–5990

Page DL and Dupont WD (1990) Anatomic markers of human premalignancy and risk of breast cancer. *Cancer* **66**: 1326–1335

Page DL, Dupont WD, Rogers LW and Rados MS (1985) Atypical hyperplastic lesions of the female breast – a long-term follow-up study. *Cancer* **55**: 2648–2708

Paine TM, Soule HD, Pauley RJ and Dawson PJ (1992) Characterization of epithelial phenotypes in mortal and immortal human breast cells. *Int J Cancer* **50**: 463–473

Pauley RJ, Soule DH, Tait L, Miller FR, Wolman SR, Dawson PJ and Heppner GH (1993) The MCF-10 family of spontaneously immortalized human breast epithelial cell lines: models of neoplastic progression. *Eur J Cancer Prev* **2** (suppl. 3): 67–76

Pitol HC and Dragan YP (1991) Facts and theories concerning the mechanisms of carcinogenesis. *FASEB J* **5**: 2280–2286

Sambrook J, Fritsch EF and Maniatis T (1989) In *Molecular Cloning, a Laboratory Manual*, 2nd edn. Cold Spring Harbor Laboratory Press: Cold Spring Harbor, NY

Soule HD, Vazguez J, Long A, Albert S and Brennan M (1973) A human cell line from a pleural effusion derived from a breast carcinoma. *J Natl Cancer Inst* **51**: 1409–1416

Soule HD, Maloney TM, Wolman SR, Peterson WD, Brenz R, McGrath CM, Russo J, Pauley RJ, Jones RF and Brooks SC (1990) Isolation and characterization of a spontaneously immortalized human breast epithelial cell line, MCF-10. *Cancer Res* **50**: 6075–6086

Toikkanen S, Helin H, Isola J and Joensuu H (1992) Prognostic significance of Her-2 oncoprotein expression in breast cancer: a 30-year follow-up. *J Clin Oncol* **10**: 1044–1048

Wright C, Nicholson S, Angus B, Sainbury JR, Farndon J, Cairns J, Harris AL and Horne CH (1992) Relationship between c-erbB-2 protein product expression and response to endocrine therapy in advanced breast cancer. *Br J Cancer* **65**: 118–121

Xie Y, Pendergast AM and Hung MC (1995) Dominant-negative mutants of Grb2 induced reversal of the transformed phenotypes caused by the point mutation-activated rat HER-2/Neu. *J Biol Chem* **270**: 30717–30724

© Cancer Research Campaign 1998